# Canine osteosarcoma in comparative oncology: Molecular mechanisms through to treatment discovery

**DOI:** 10.3389/fvets.2022.965391

**Published:** 2022-12-08

**Authors:** Siobhan Simpson, Albert A. Rizvanov, Jennie N. Jeyapalan, Simone de Brot, Catrin S. Rutland

**Affiliations:** ^1^Faculty of Medicine and Health Sciences, School of Veterinary Medicine and Science, University of Nottingham, Nottingham, United Kingdom; ^2^Institute of Fundamental Medicine and Biology, Kazan Federal University, Kazan, Russia; ^3^Faculty of Medicine and Health Science, Biodiscovery Institute, University of Nottingham, Nottingham, United Kingdom; ^4^Comparative Pathology Platform (COMPATH), Institute of Animal Pathology, University of Bern, Bern, Switzerland

**Keywords:** bone cancer, canine, genes, human, osteosarcoma, protein, treatment

## Abstract

Cancer is a leading cause of non-communicable morbidity and mortality throughout the world, similarly, in dogs, the most frequent cause of mortality is tumors. Some types of cancer, including osteosarcoma (OSA), occur at much higher rates in dogs than people. Dogs therefore not only require treatment themselves but can also act as an effective parallel patient population for the human disease equivalent. It should be noted that although there are many similarities between canine and human OSA, there are also key differences and it is important to research and highlight these features. Despite progress using chorioallantoic membrane models, 2D and 3D *in vitro* models, and rodent OSA models, many more insights into the molecular and cellular mechanisms, drug development, and treatment are being discovered in a variety of canine OSA patient populations.

## Introduction

In both human and canine patients the predominant bone cancer diagnosis is OSA (1, 2). Sarcomas are tumors originating in tissues derived from the mesoderm, affecting bone, cartilage and connective tissue (3). Osteosarcoma produces malignant bone or osteoid tissue, but a unifying feature is that all types of OSA histologically produce tumor osteoid (4). Archetypal OSA consists of a primary tumor, usually originating within the medullary cavity and spreading to the surface of the bone, but they can be extra-osseous (5). Typically the tumor grows, proliferates, invades, and left unchecked frequently metastasises to the lungs (6). OSA subtypes include osteoblastic (bony), chondroblastic (cartilaginous), and fibroblastic (resemble atypical fibroblasts), with a range of rare types, and those not originating in the medullary cavity (5), see also a previous review (7).

### Overview of experimental OSA models

Rodent and chorioallantoic membrane (CAM) models have been utilized in OSA studies, in addition to a variety of *in vitro* methods, however each has a number of limitations. Early rodent models represent OSA well-histologically, but do not represent the true etiology of the disease (8). Immunocompromised mice inoculated with human OSA cell lines or grafts have served well for studying metastasis, drug screening, and helped toward identifying activation pathways, but have limited capacity in understanding OSA development and immune system interactions, although advances in this area are continuing (9–11). P53 and Rb mutation transgenic mouse studies have an overall relatively high cost and difficulties relating to breeding and development of non-OSA cancers, but have shown similarities to the human disease (12–15).

Although CAM models have been used for over a century, an avian environment does not always replicate the mammalian tumor environment or immune system. Although angiogenesis in this model can assist with looking at invasion, drug development, and metastasis, it has not been widely used for OSA models (16, 17). Indeed many of the models developed for OSA failed to produce a tumor and/or osteoid, a key component of OSA (18). The advancements in 3D *in vitro* models over 2D ones, represents a step forward in understanding microenvironment interactions and mechanisms, with fewer limitations than traditional culturing models (19, 20). Examples using liquid overlays (21) and ultra-low binding plates to develop spheroid formation have been used, the latter helping identify a potential role of miR-335 in OSA (21). Hanging drop methods have also been used, especially alongside 2D cell cultures to investigate VEGF expression, vital for angiogenesis (22).

Unfortunately, none of these methods perfectly recreate the tumor microenvironment, or replicate growth and development of the cancer. While these methods have improved prevention, diagnosis, and treatment of a range of diseases, OSA cure rates and survival times have not improved significantly in decades (23, 24). What is really required is a model or parallel patient population that accurately recapitulates the clinical, biological and molecular aspects of human/pediatric OSA.

### OSA in dogs and people–parallel patient populations

Given the spontaneous nature of OSA in dogs, and the clinical relevance of canine to human OSA, these natural models might be better described as parallel patient populations (7, 25). Naturally occurring parallel patient populations allow researchers access to additional cases of disease without inducing disease.

Current understanding of OSA disease processes and treatments is largely based on studying affected individuals compared to unaffected individuals, or assessing differing types of OSA, with computer simulations/bioinformatics playing an increasing role (26, 27). The development and progression of OSA is frequently influenced by a combination of environmental and genetic risk factors. Understanding the basis of disease and development of new treatments *via* animal models, particularly within naturally occurring animal populations, is crucial, however care must be taken to ensure phenotypes are representative of the disease.

The overall canine population is genetically heterogeneous, however breeds can be comparatively homogeneous which further enhances their value for comparing genetic mechanisms of disease (28). Some breeds are at increased risk of developing OSA (1), making them a valuable parallel patient population. Human diseases may progress over a number of years, and spontaneously occurring canine OSA reflects this progression in contrast to laboratory models which are often investigated over much shorter periods of time. Indeed many human disease phenotypes are closely matched to canine disease phenotypes, exhibiting similar pathologies, progression, treatment options, and prognosis (29–31), this includes OSA (7). The canine and human OSA biological and histological similarities, alongside treatment trials and comparisons have been evidenced through numerous studies across the decades. More recently, the molecular and cellular comparisons undertaken between the two species, as detailed within this review, have provided crucial steps toward understanding both the limitations and benefits of studying canine OSA as a parallel population.

### Similarities and differences in OSA incidence, risk factors and survival rates between people and dogs

Dogs naturally have a higher OSA incidence than people. Human population studies have shown there are roughly 0.89 cases of bone cancer per 100,000 people/annum (32, 33). In a population of 394,061 insured dogs, 764 (0.19%) developed a bone tumor (1), representing an incidence rate of 27.2 dogs per 100,000/annum, a much higher rate than in people. The higher incidence rate of canine OSA makes the pet dog population an ideal parallel patient population for investigating the disease in humans. In people, there is increasing evidence of variation in the incidence rate between families and different populations (2, 34, 35). Interestingly, OSA in dogs is highly influenced by breed, with Irish wolfhounds displaying the highest levels (12.3% of the population), with some other breeds mostly unaffected (1, 36).

Osteosarcoma is bimodal in people peaking in the young (< 20 years old) and elderly (>60 years old) (2, 32, 33, 37, 38). Although widely reported as a bimodal occurrence in canines, with peaks at 1.5–3 and 7–9 years, this bimodal observation has not been shown all studies (39–43). An additional important difference between the species is that OSA is more prominent in the older dog range (7–9 years), whereas in people the incidence is highest within the pediatric population. The first cross-species genomic analysis between canine and human OSA indicated that there were very strong gene expression similarities between the two species (25). Hierarchical clustering showed branching between OSA and normal tissues but showed no distinct branching of canine and human OSA. This study specifically compared pediatric tumors from children against canine (ages not stated) OSA and was able to draw the conclusion regarding the similarities between canine and pediatric OSA but did not test adult human tumors (25). Notably a later study looked at both juvenile and adult canine tumors indicated that the adult dog was a good model regarding genomic features and clinical characteristics (44). This supports the data indicating clinical presentation and diagnosis, histological presentation and treatment similarities between canine and juvenile human OSA (45, 46).

Despite a general trend of improving 5-year-event-free-survival rates across all cancer types in people (24, 47), OSA has not shown comparable improvements in mortality rates (2, 47, 48). The 5-year-event-free-survival for individuals with metastatic tumors at diagnosis was reported to be 27.4%, increasing to 70% in individuals with no metastases at diagnosis (2, 6). The 1-year survival rate for canines is typically < 45% (49–51). It is worth noting that for appendicular OSA, the 1 and 2-year survival rates have been published at just 11.5 and 2%, respectively for dogs receiving amputation only as a treatment option (52). These similarities in presentation not only support the rationale for the dog as a parallel patient population for studying OSA but also highlight the urgent need to develop improved treatments and cures.

The common risk factors associated with OSA development in both humans and canines include sex, growth, puberty (2, 34, 53), in addition to population/breed and a range of molecular associations. Growth has been associated with the development of OSA in both people and dogs (1, 36). In people, age of onset frequently coincides with rapid bone growth during puberty, tumor sites are most frequently situated at the end of bones where active growth occurs (2). In canines there is not as much evidence linking to growth, given the later onset of OSA in general, however OSA predominantly occurs in weight-bearing bones and adjacent to late-closing physes (1, 36). Large dog breeds make up the majority of canine OSA cases reflecting the human population where affected individuals are more likely to be taller than average (1, 34, 54).

In the canine population, as with the human population, there appears to be a skewed sex ratio with males typically more affected by OSA, and at younger ages, than females (1, 2, 32, 34, 55). Additionally, neutering status, although less relevant in the human context, appears to contribute to risk with neutered dogs more likely to develop OSA than non-neutered counterparts (36). The neutering effect, combined with the association with puberty, indicates that sex hormone signaling may play complex roles in OSA.

Presently there are over 544 canine “potential models for human traits” listed in OMIA (Online Mendelian Inheritance in Animals), more than any other species (29, 30). Dogs are typically treated as family members and so inhabit the same environment as their owners, alongside many of the environmental and other risk factors impacting disease risk, initiation and progression. Pet dogs also frequently benefit from high quality medical care, such that illnesses are detected and treated promptly, similar in a way to people (56). This also means that the amount of information being collected by veterinary clinics, researchers, and insurance companies expands the data available. These canine population characteristics represent a valuable resource for modeling human disease. Although understanding diseases and developing novel treatments in companion animals exhibiting occurring disease is less contentious than inducing disease in experimental animals, ethical concerns regarding treatment of individuals and gaining informed consent from owners remain (57).

### Similarities and differences between OSA molecular mechanisms in people and dogs

Developing new treatments is expensive and time consuming. Only 4.1% of potential new compounds progress from preclinical discovery to patient use, taking on average 13.5 years (58, 59). In order to create targeted pharmaceuticals in shorter time frames, understanding the genetic mechanisms behind diseases are critical (60). Indeed, parallel animal patient populations of disease, including OSA, play crucial roles in identifying genetic loci associations and biomarkers, which may lead to target identification, to help determine appropriate drugs, leading through to target validation (61–63). Much of the molecular work, underpinning early drug development and repurposing, is facilitated by the conservation of many fundamental biological pathways between species (64–66).

Pedigree breeds in dogs are generally fairly closed populations, ancestry can often be traced for many generations, and even back to the breed's founding members (28, 67, 68). Although this restricts genetic diversity within breeds, it facilitates understanding the mode of inheritance of traits and diseases (67). Both the founder effects and later inbreeding within canine pedigree breeds have led to divergent allele frequencies between breeds, resulting in some breeds exhibiting higher disease frequencies (28, 69). As a result, differing breeds have homologs of numerous human conditions, making them ideal for identifying potential genetic loci associated with disease for both canine and human benefit.

Some cases of human OSA have been associated with heritable cancer syndromes, and the genetic basis of these has been established (70–72). Despite this, most human OSA cases are not considered to be heritable. Some somatic mutations in tumor suppressor genes have been identified in individuals with heritable cancer syndromes such as Li-Fraumeni syndrome, and other mutations have been identified in OSA tissue compared to normal tissue (70, 73–75). Interesting, to date, only two somatic genetic mutations have been specifically associated with OSA (53). Despite the lack of heritability and somatic genetic mutations, over 900 genes are associated with human OSA (76). These associations have been identified due to differences in expression, or identification of mutations, that have arisen in the tumor compared to the non-tumor tissue (77–79). Mutations within OSA tumor tissue may exist as a cause, or as a result, of the tumor. Differential expression, and mutations, may also exist *via* genomic and chromosomal instability, which in itself is a reported factor in many types of cancer progression (80, 81). Osteosarcoma in people has been shown to display chromosomal instability associated with mutations in the *TP53* gene (82). Aneuploidy can occur as a consequence of chromosomal instability, which can lead to the gene overexpression in affected malignant cells, causing disruption to the normal cell processes (83). Although mutations in *TP53* look likely to be associated with chromosomal instability, the gene itself is not over expressed following aneuploidy (82, 83). TP53 has also been implicated in canine OSA with whole genome sequencing and whole exome sequencing (WES) indicating frequent *TP53* mutations in canine OSA tumors, at rates of up to 83%, specific mutation rates were variable between breeds (44, 84, 85). *TP53* mutations have featured heavily in many of canine OSA studies, however findings still differ between these studies overall. For example it was found that *TP53* missense mutations in dogs who had amputation followed by chemotherapy were associated with a longer DFI than wild type of null tumor samples investigated (85). Although similar results have not yet been observed in human OSA, other cancer types and mutant cell lines have shown improved treatment responses (86, 87).

Gene expression following treatment has also highlighted key similarities between people and dogs. Studies identifying gene expression in canine patients responding, and not responding, to chemotherapy treatment, were later found to be similar in people, indicating the value of the dog as a parallel patient population for human OSA (88). It should also be noted that gene expression variations have been observed on some occasions, despite the often-high similarities in many other studies (25), thus indicating a potential limitation of canine comparisons with human OSA.

In canine OSA patients, 33 loci have been associated with the disease across three breeds, and an additional single locus is associated in Deerhounds (89, 90). None of these loci are consistently associated across breeds, suggesting there may be a difference between breeds regarding genetic predisposition to developing OSA (89, 90). In addition to the 34 genetic loci identified, genes have been identified as differentially expressed in canine OSA compared to non-tumor tissue, many of which have implications for growth and metastasis, and are potential drug targets (55, 91–96). These genes have been identified utilizing canine OSA tumor tissue, and/or canine OSA cell lines. Some proteins of interest have also had histological work undertaken to start identifying their presence and relevance in OSA (55, 95). There has also been variation in the expression of genes within tumors associated with survival time in canine OSA (97–100).

### Shared genes and proteins of interest for development of future treatments

In both humans and dogs, effective treatment for OSA involves surgery to remove primary tumors 27, 32], often combined with neoadjuvant and/or adjuvant radiotherapy and chemotherapy [33, 34]. The type of surgery rarely has an impact on survival for most human tumors [27], more important prognostic factors are how the tumor responds to chemotherapy and the presence of metastases prior to surgery [27]. In order to advance the treatments available, genomics and drug discovery are providing potential new treatments. Increasingly, comparisons between results from human and canine OSA studies are showing shared genes of interest between the two species. Many of these studies also highlight the need for further testing in relation to potential therapeutic agents.

**Figure 1:**
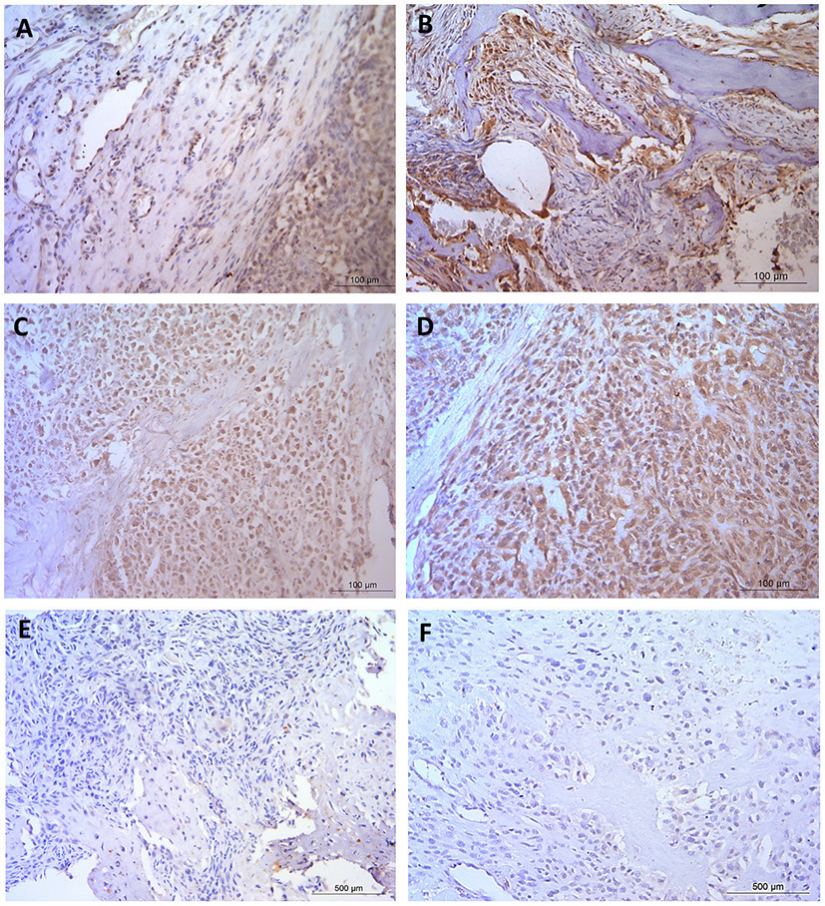
Immunohistochemical staining in naturally occurring canine osteosarcoma tissue. **(A,B)** GLUT1 and **(C,D)** MMP3, both staining patterns expressing positive nuclear, cytoplasmic and vascular tissue with negative staining observed in the osteoid. **(E,F)** Negative control. Scale bars represent **(A–D)** 100 μm, **(E,F)** 500 μm. Staining was conducted with ethics, techniques and tissues as previously published (95).

Comparative transcriptional profiling of dogs and human OSAs has highlighted the similarities between the tumor tissues in the two species. One example was OSA tissue cluster analysis undertaken on 265 orthologous transcripts on pediatric human OSA compared to canine (age not stated) OSA (25). The conclusion was that it was not possible to differentiate between canine and pediatric human OSA tissues yet normal tissues from both species did branch (25). Similar outcome predictions for specific genes in both humans and dogs were also observed. Examples of these include interleukin-8 (IL-8) and solute carrier family 1 (glial high affinity glutamate transporter), member 3 (SLC1A3). Increased expression levels of IL-8 and SLC1A3 predicted poor clinical outcomes in tissues from both species, a result initially identified in canine samples, then followed up and confirmed using human OSA data and both human and canine OSA cell lines (25). Interestingly, increased expression of *SLC2A1* (GLUT1) within tumors also resulted in poorer prognosis and a shorter disease free interval in people (101). *SLC2A1*/GLUT1 (see Figure 1) levels were also significantly increased in naturally occurring canine OSA tissue compared to normal bone tissue (55, 95). Inhibition of SLC2A1 and cellular glucose transport has been achieved by a number of pharmaceuticals, however, as with the MMP3 inhibitors, these have yet to be utilized in OSA trials in either people or dogs (102–105). Monoclonal IL-8 antibody therapy could also be of interest given the importance of this chemo-attractant angiogenic factor, which has been implicated in a number of cancers (106, 107). Although not yet tested in OSA, clinical trials using IL-8 monoclonal antibodies in other cancers types are ongoing (108) and provide an interesting target given the links between increased IL-8 expression and doxorubicin resistance (109, 110).

Another good example of comparative OSA highlighted the role of MMP3, with increased expression linked with a poor prognosis in OSA, and to formation of metastases (23, 111). Tsai et al. (112) and Huang et al. (113) identified higher expression of *MMP3* in human OSA compared to normal bone. Additionally Adiguzel et al. (114) reported on MMP3 polymorphisms associated with OSA in people. Naturally occurring OSA was also associated with increased *MMP3* levels in canine patients (55), and work was later undertaken to show expression patterns (Figure 1) of the protein in tissue (95). Despite the increasing evidence regarding MMP3, neither the selective inhibitor of MMP3 (UK370106) (115) or the generic MMP inhibitor (marimastat) (116) have been assessed in relation to restricting primary tumors or metastatic tumor growth in canine or human OSA, despite some trials in other tumor types.

The Dickkopf proteins are differentially expressed in a number of cancers, and inhibit Wnt signaling which, in turn, is aberrant in many cancers (117–119). Reduced *DKK3* expression in human breast, endometrial, and cervical cancer, has implicated it as a tumor suppressor (120–123). *DKK3* expression within OSA has resulted in conflicting reports. In human OSA cell lines, and in xenograft mice, *DKK3* expression was reduced, however subsequent restoration of *DKK3* expression resulted in reduced tumor and metastatic growth (124). In contrast, *DKK3* was more highly expressed in human OSA cells overexpressing *NKD2* and in tumor tissue (125), and also in tumor tissue compared to non-affected bone in naturally occurring canine OSA (55). Despite differences compared to some cancers, this outcome agreed with *DKK3* knockdown in cells overexpressing *NKD2* which exhibited increased proliferation, indicating a possible mechanism of *NKD2* induced metastasis, although the authors noted more work into the mechanisms was required (125). With a lack of drugs available acting on *DKK3*, development in this direction could prove useful for OSA in both people and dogs. Although these examples represent just a small number of the genes and proteins of interest in both human and canine OSA, it helps show the benefits of using the dog as a parallel patient population for this cancer, especially in relation to drug development. Table 1 provides a summary of the genes, proteins and pathways detailed in this review.

**Table 1:** Summary of key comparative genes, proteins and pathways in human and canine osteosarcoma.

**Gene/Protein/Pathway**	**Human**	**Canine**
*TP53*	Chromosomal instability associated with mutations in the TP53 gene (82). Chromosomal abnormalities leading to aneuploidy, resulting in overexpression, disrupted cell processes (83).	Frequent TP53 mutations in canine OSA (44, 84, 85).
*TP53, RB1, MYC, PTEN, RUNX2, CDKN2A, CDKN2B*	Copy number aberrations (126–128).	Copy number aberrations (126–128).
*DMD*	Somatic DMD variants found in 5/8 patients (44).	*DMD* aberrations, including canine specific mutations, in 50% specimens (44).
*SETD*	Dysregulation of SETD2 implied in human OSA (25, 101).	Putatively inactivating somatic *SETD2* in 42% of specimens including some canine specific compared to human (44).
IL-8 and SLC1A3	Increased expression levels of IL-8 and SLC1A3 predicted poor clinical outcomes (25). Increased expression of *SLC2A1* (GLUT1) within tumors resulted in poorer prognosis and a shorter disease free interval (104).	Increased expression levels of IL-8 and SLC1A3 predicted poor clinical outcomes (25). Increased *SLC2A1*/GLUT1 levels in OSA tissue compared to normal bone tissue (55, 95).
MMP3	Higher expression of *MMP3* in OSA tissue compared to normal bone (115, 116). MMP3 polymorphisms associated with OSA (117). Increased expression linked with a poor prognosis and to formation of metastases (23, 114).	Increased *MMP3* levels associated with OSA (55), proteins expression shown in OSA tissue (95).
DKK3	*DKK3* expression reduced in OSA cell lines, but subsequent restoration of *DKK3* expression resulted in reduced tumor and metastatic growth (129). In contrast, *DKK3* was more highly expressed in OSA cells overexpressing *NKD2* and in tumor tissue (130),	*DKK3* expression increased in OSA tissue compared to non-affected bone (55).
PI3K, P13K-Akt, P13K/mTOR and MAPK pathway mutations	PI3K/mTOR shared vulnerability for both species (131)	Mutations in PI3K in 37% of the samples and 17% for MAPK (44). Dysregulation of P13K-Akt pathway and *COL6A3, COL5A2, TNC, and ITGB5* activation (103, 132), and PI3K/mTOR (131)

Whole genome and exome sequencing have also discovered not only where mutations within pathways such as PI3K and MAPK are similar between human and canine OSA, but have also identified novel aberrations in canines, such as those in *SETD* and *DMD*, which have not yet been reported in people (44). Although these aberrations have not yet been found in human OSA cases, despite the high sequence homology between the two species, it is known that dysregulation of SETD2 has been implied in human OSA (25, 133). Although the *DMD* gene encoding dystrophin is more commonly associated with Duchene and Becker muscular dystrophy in both species, other studies have shown somatic *DMD* variants in human OSA patients (129). Comparative canine and human transcriptomic studies have also identified annotations and pathways unique to particular cancers. For example, annotations unique to bone material synthesis, including *COL5A2, COL6A3*, and *COL12A*, were discovered in OSA in both species but were not present in melanoma, pulmonary carcinoma, or B- and T-cell lymphoma (130). Considerable insights into possible pathways and biomarkers can be provided by such studies. Often potential biomarkers, or targets that have known chemistries presently available, including examples such as *COL16A1* and *KDELR2* (130), are highlighted as areas needing more research.

### Shared MicroRNAs in comparative studies

Recent reviews outlining the potential comparative values for investigating vasculogenic mimicry molecular pathways and microRNAs (miRNA) in the dog, highlight how little work has been conducted in this species compared to humans (134, 135). They contain detailed discussions around miRNAs and lncRNAs and provide interesting reading around these areas, especially in relation to vasculogenic mimicry in canines in comparison to people, which is not therefore covered in the present review. In both dogs and people decreased expression of miR-1, miR-133b and miR-196A have been shown to be involved in proliferation-invasion, miR-34 with proliferation and 14q32 locus (including mir-544, miR-396-3p, miR134 and miR-382) with proliferation-apoptosis in OSA (135). Additionally increased miR-9 has been associated with invasion and increases in miR-106b cluster have been associated with proliferation (135). Comparative examples such as the dysregulation of the 14q32 miRNA cluster in both dogs and people, not only identified a potentially conserved mechanism related to the aggressive and invasive biological behavior of OSA in both species, but yet again emphasize the similarities between the two species (136). Another example is the discovery of miR-1 and miR-133b which showed lower expression levels in canine OSA compared to normal tissue, yet increased expression of their targets MET and MCL1 (137). Interestingly a previous study had shown that both miR-1 and miR-133b were differentially expressed in human OSA affected tissue compared to non-OSA bone (138). MiR-34a looks especially promising given its links to both human and canine OSA, its anti-proliferation and metastasis inhibition activities and the research relating to a genetically engineered pre-microRNA-34a prodrug (139–141). Work published after the recent review (135) compared 19 miRNA candidates expressing differential expression in OSA samples compared to non-affected tissue in both people and dogs were also assessed (142). This research showed that expression miR-223 increases and in let-7b and miR-130a decreases were associated with increased risk and a shorter disease free interval. These were highlighted as potential targets and/or biomarkers for OSA.

### Translational drug development studies

In addition to the molecular studies highlighting potential targets of interest, a number of canine OSA trials have assessed treatment regimens which were primarily designed to increase survival times. Early evidence showed that while amputation alone increased canine survival by around 2.5 months, addition of liposome-encapsulated muramyl tripeptide (L-MTP-PE) administration following amputation prolonged survival by an additional 5 months, primarily by reducing OSA metastasis development (143). Later randomized canine trials showed the outcomes of combining differing protocols of L-MTP-PE and cisplatin chemotherapy treatment. L-MTP-PE exhibited antimetastatic activity when administered post amputation, increasing survival times to around 14.4 months when cisplatin was administered after L-MTP-PE (144). The survival advantages observed following L-MTP-PE alone were not observed when cisplatin and L-MTP-PE were administered concurrently rather than sequentially, indicating that treatment timing is crucial. Trials of this drug in children with OSA revealed an 8% improvement in survival (145), but anti-tumor effects and increased survival times were also noted when treating human OSA patients with L-MTP-PE, especially when chemotherapy was administered (146–148). For example, a 24-week treatment with L-MTP-PE increased median time to relapse from 4.5 months for the control group to 9 months for the treatment group (146, 147). It was also noted that plasma levels of cytokines including IL-8, TNF-α and IL-6 reduced following treatment, all of which may play roles in monocyte-mediated tumor cell death (146, 147). This work followed the smaller phase II trial indicating histological changes to pulmonary metastases in OSA patients (148).

HER2/neu, a tyrosine kinase receptor within the epidermal growth factor receptor family, is expressed in osteosarcoma stem cells (149). Expression has been found in 40% of pediatric and canine osteosarcoma, and associated with higher metastatic rates, reduced response to neoadjuvant chemotherapy, and reduced survival times (150–152). A chimeric human HER2/neu fusion protein (ADXS31-164, also now known as ADXS-HER2 and OST-HER2) was tested in dogs with a histopathological and immunohistochemical diagnosis of HER2/neu OSA, following amputation/limb sparing surgery and treatment with carboplatin (153). Disease-free interval (DFI) following the intervention was 615 days, median survival time (MST) was 956 days, and overall survival rates at 1, 2, and 3 years were 77.8, 67, and 56%, respectively. The authors noted significant outcome improvements compared to matched historical control group rates showing a DFI of 123–257 days, a MST of 207–321 days, and overall survival rates of 35.4% (1 year) and 10–15% (2 years). Additionally, this study showed only mild side-effects of ADXS31-164 when administered to canine patients. This therapy specifically induced HER2-specific immunity, targeting the cells expressing HER2/neu, broke peripheral tolerance to HER2/neu and mediated cytotoxic T-cell–dependent tumor regression (153). In 2016, ADXS-HER2 was granted orphan-drug designation, then rare pediatric disease designation in 2021, from the FDA and EMA, for the treatment of OSA. In 2021 ADVAXIS Immunotherapies, in collaboration with the Children's Oncology Group, reported that the first human OSA patient had received doses in the Phase IIb trial of this drug (154). The outcomes from this trial, including any clinical results and mechanistic studies will be of great interest regarding not only human and canine OSA, but in relation to other cancer types which also express HER2 including mammary carcinoma (126).

The angiotensin-receptor blocker losartan, when used in combination with the kinase inhibitor toceranib, has also shown promising results in canine OSA patients (127). By blocking OSA-elicited monocyte recruitment *via* the action of losartan inhibiting the CCL2–CCR2 axis, clinical benefits including tumor stabilization and/or regression were observed in half of the dogs. Notably, both human and canine OSA cells secrete CCL2, resulting in monocyte migration. By interrupting the CCR2–CCL2 axis and by blocking monocyte migration, these trials have provided more insights into the tumor microenvironment and indicated a direct mechanism by which these therapeutic agents could work in human OSA patients. Owing to the success of this canine OSA trial published in 2021, a phase I clinical trial (NCT03900793) was initiated in pediatric and young adult OSA patients with lung metastases.

## Limitations of canine OSA models

One of the limitations of this area of research is that frequently the research concentrates on either dogs or people with relatively few comparisons of the two using the same analysis and techniques. Although this individual species specific research is required, the number of directly comparative studies is much lower and makes comparative conclusions more complex. This also complicates matters with regards to potential differences observed between breeds and age of onset, as highlighted in this review. For example although the differences between breeds are often presented, in many cases the comparisons between each of the breeds and human OSA are not frequently investigated. Canines are often referred to as a good model for juvenile human OSA but in addition to the published comparisons between general canine OSA and juvenile human OSA, it must be highlighted that many studies do draw any conclusions regarding juvenile or later onset OSA specifically in either species. In some OSA studies, particularly the canine studies, the ages of the patients are not presented or the juvenile/later onset differences, if any, are not specifically referred to or investigated. Additionally, particularly when thinking about juvenile OSA, matters such as whether growth is a risk factor is not as easy to justify in dogs compared to people.

With molecular differences between OSA samples differing between individuals within the same species, it is natural to expect differences between the species and between the ages of the individuals. When comparing canine OSA to human pediatric OSA in particular, mutational burden must be considered. Generally pediatric human cancers present with fewer mutational burdens, compared with geriatric tumors [as reviewed previously (128)], therefore this must be considered when comparing against older canines with pediatric OSA. There are concomitant arguments for using the dog as a model of aging (also presenting with limitations and differences) (131). Although similarities between canine and human DNA repair machinery have been shown, such as in lymphoma, mammary tumors and even OSA (132, 155, 156), not every mechanism may be similar, for example base excision repair and nucleotide excision repair have both been shown to be lower in canines (157). Unfortunately little is known about DNA repair in canine OSA (158) even though it may play significant roles when comparing geriatric with juvenile/pediatric OSA. This further accentuates the need for vigilance when researching OSA in general including both OSA specific molecular mechanisms and pathways, and those related to more general factors such as aging.

## Discussion

Canine OSA occurs naturally within the population, reflecting the development of human OSA (7, 159). In contrast animal models of OSA rely on chemical induction, xeno/allografts, and genetically engineered animals which are unlikely to reflect many aspects of naturally occurring disease (159–162). Canine OSA has several features that can accelerate the understanding of the molecular basis of OSA, potentially facilitating more rapid development of novel diagnostic and therapeutic targets relevant to both people and dogs. The advantages of canine OSA parallel patient populations include a shared environment with people, natural disease progression, higher incidence rates, alongside shorter lifespans resulting in a quicker clinical course. Arguments have also been put forward that in addition to the dog being a good parallel patient population for OSA in people, the reverse is also technically true. It has also been indicated that canine OSA may represent a more accelerated biology than human OSA and that novel metastasis-associated tumor targets may be more readily identifiable in canine tissues (25). Whilst this review has concentrated on some of the shared molecular observations and mechanisms, there are many examples presented where canines do not exactly mirror human OSA. Canine OSA parallel patient populations can therefore give valuable insights, advancing knowledge about disease progression and development, cellular and molecular mechanisms, and therapeutic and treatment strategies, in both people and dogs.

## Author contributions

CR developed the concept, designed and supervised the manuscript, and created Figure 1. SS and CR drafted the manuscript. CR and AR received funding. All authors revised the manuscript critically, contributed intellectually, and approved the submitted version.
